# Differentiation of Breast Cancer from Fibroadenoma with Dual-Echo Dynamic Contrast-Enhanced MRI

**DOI:** 10.1371/journal.pone.0067731

**Published:** 2013-07-02

**Authors:** Shiwei Wang, Zachary DelProposto, Haoyu Wang, Xuewei Ding, Conghua Ji, Bei Wang, Maosheng Xu

**Affiliations:** 1 Department of Radiology, First Affiliated Hospital of Zhejiang Chinese Medical University, Hangzhou, Zhejiang, China; 2 Department of Radiology, Henry Ford Hospital, Detroit, Michigan, United States of America; 3 Beijing Key Laboratory of Medical Physics and Engineering, Peking University, Beijing, China; 4 Clinical Evaluation and Analysis Center, First Affiliated Hospital of Zhejiang Chinese Medical University, Hangzhou, Zhejiang, China; 5 Department of Breast Disease, First Affiliated Hospital of Zhejiang Chinese Medical University, Hangzhou, Zhejiang, China; Wayne State University, United States of America

## Abstract

Dynamic contrast-enhanced magnetic resonance imaging (DCE MRI) of the breast is a routinely used imaging method which is highly sensitive for detecting breast malignancy. Specificity, though, remains suboptimal. Dynamic susceptibility contrast magnetic resonance imaging (DSC MRI), an alternative dynamic contrast imaging technique, evaluates perfusion-related parameters unique from DCE MRI. Previous work has shown that the combination of DSC MRI with DCE MRI can improve diagnostic specificity, though an additional administration of intravenous contrast is required. Dual-echo MRI can measure both T_1_W DCE MRI and T_2_*W DSC MRI parameters with a single contrast bolus, but has not been previously implemented in breast imaging. We have developed a dual-echo gradient-echo sequence to perform such simultaneous measurements in the breast, and use it to calculate the semi-quantitative T_1_W and T_2_*W related parameters such as peak enhancement ratio, time of maximal enhancement, regional blood flow, and regional blood volume in 20 malignant lesions and 10 benign fibroadenomas in 38 patients. Imaging parameters were compared to surgical or biopsy obtained tissue samples. Receiver operating characteristic (ROC) curves and area under the ROC curves were calculated for each parameter and combination of parameters. The time of maximal enhancement derived from DCE MRI had a 90% sensitivity and 69% specificity for predicting malignancy. When combined with DSC MRI derived regional blood flow and volume parameters, sensitivity remained unchanged at 90% but specificity increased to 80%. In conclusion, we show that dual-echo MRI with a single administration of contrast agent can simultaneously measure both T_1_W and T_2_*W related perfusion and kinetic parameters in the breast and the combination of DCE MRI and DSC MRI parameters improves the diagnostic performance of breast MRI to differentiate breast cancer from benign fibroadenomas.

## Introduction

Magnetic resonance imaging (MRI) has become an important technique for breast cancer detection, diagnosis, and staging [1]. When lesion morphology is combined with the dynamic analysis of contrast kinetics within breast lesions, the overall sensitivity of MRI is nearly 90%, and specificity varies between 67% and 72% [2], [3]. Compared to all other imaging techniques (including ultrasonography and mammography), the negative predictive value of MRI remains the highest of all modalities [4], [5].

Conventional dynamic contrast enhanced MRI (DCE MRI) is the most widely used and clinically validated technique for breast cancer MRI. It not only provides morphological information, but also typically uses high spatial resolution to estimate T_1_W-related contrast uptake parameters. Despite high sensitivity, diagnostic specificity remains unsatisfactory [6], [7]. An alternative dynamic contrast technique, known as dynamic susceptibility-contrast MRI (DSC MRI) uses high temporal resolution to obtain perfusion-related parameters based on T_2_*measurements, such as relative regional blood volume (rBV)and relative regional blood flow (rBF) [8]–[10]. Perfusion-related parameters can differentiate malignant from benign lesions [8]–[10]. The combination of conventional DCE MRI and DSC MRI with two administrations of contrast agent (CA) has demonstrated the capability to substantially improve the diagnostic specificity of breast MRI [7], [11].

Using dual-echo MRI, and with a single administration of contrast agent, T_1_W and T_2_*W related measurements can be simultaneously acquired, with the first echo acquiring T_1_W (DCE MRI) data and the second echo (6.3 ms later) acquiring T_2_*W data (DSC MRI)[12]–[14]. To date, dual-echo MRI has not been performed in the breast. We have developed a dual gradient echo (GRE) sequence and have used it to evaluate both T_1_W and T_2_*W related parameters in differentiating breast cancer from the most common benign breast mass, fibroadenoma.

## Methods

### Ethics Statement

The study was approved by the ethics committee of the First Affiliated Hospital of Zhejiang Chinese Medical University, Hangzhou, China, and performed in accordance with the ethical guidelines of the Declaration of Helsinki. After a throughout explanation of the study to the patient, written informed consent was obtained from 37 patients and 1 guardian on the behalf of the minor.

### Patients

Between May 2011 to March 2012, 46 patients with suspected breast cancer based on mammography and the BIRADS category being 4 or 5 underwent dynamic contrast enhanced MRI and dual-echo dynamic contrast enhanced MRI examination. Of the 46 patients, 38 patients had subsequent surgery or biopsy with pathologic correlation (mean age: 45 years, range: 15–65 years).There were 45 lesions evaluated in 38 patients, with 5 patients having two lesions and one patient having three lesions. In patients with multiple lesions, only the largest lesion was analyzed in this study. All patients had normal renal function (eGFR>60 ml/min/1.73 m^2^).

### Dual-Echo based DCE MRI and DSC MRI

A dual-echo based gradient echo sequence has been implemented to acquire both DCE MR and DSC MR images simultaneously with the 1st echo as T_1_W and the 2nd echo as T_2_*W. The signal intensity of a GRE can be given by
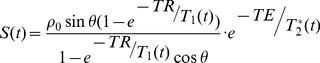
(1)Where, *ρ_0_* denotes the spin density, *θ* denotes the flip angle, *TR* denotes the repetition time, *TE* denotes echo time, *T_1_(t)* denotes the longitude relaxation time at time t. The equation clearly shows that the signal of DCE MR and DSC MR images are determined by not only T_1_ component, but also the T_2_* effect. That is to say, there are errors associated with the commonly used CK model for perfusion which assumes a constant T_2_*, and the commonly used CK model for permeability which assume a constant T_1_. Simultaneously acquired T_1_W and T_2_W* data make it possible to correct the errors.

To take into account the T_2_* effect in permeability analysis, we first calculate T_2_* map by taking the advantage of the dual echo pulse sequence using Equation (2),
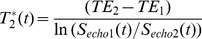
(2)Where *S_echo1_(t)* and *S_echo2_(t)* are signal intensities from the 1st echo (*TE_1_*) and 2nd echo (*TE_2_*), respectively. To exclude T_2_* dependency in each pixel, the signal intensity *S(t)* of Equation (1) is divided by the corresponding T_2_* exponential component thereby obtaining only T_1_-dependent signal intensity *S_T1_(t)* for T_1_W DCE MR imaging,



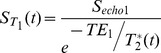
(3)To take into account the T_1_ effect on perfusion, we take the advantage of the dual-echo sequence to calculate only T_2_*-dependent signal intensity S_T2*_(t) using Equation (4),
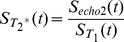
(4)


### MR Imaging Technique

All imaging was performed on a 3T whole-body MRI scanner (Verio, Siemens, Germany) with a sixteen-channel phased-array breast coil. Data were obtained for routine clinical diagnosis, which included routine clinical semi-quantitative DCE MRI in addition to the experimental dual-echo DCE/DSC MRI sequence.

Before injection, the following sequences were acquired: transverse Turbo Inversion Recovery Magnitude (TIRM) (TR = 4000 ms, TE = 70 ms, slice thickness = 4 mm, FOV = 340 mm×340 mm, average = 2, number of slices = 34, acquisition time = 168 s) and transverse diffusion weighted imaging (TR = 7000 ms, TE = 85 ms, slice thickness = 4 mm, FOV = 340 mm×340 mm, average = 3, number of slices = 24, acquisition time = 210 s).Then, a transverse dynamic contrast-enhanced (DCE) T_1_-weighted gradient-echo sequence(3D FLASH)(TR = 4.51 ms, TE = 1.61 ms, flip angle = 10°slice thickness = 1 mm, FOV = 340 mm×340 mm, average = 1,slab = 1, number of time points (measurements) = 7, acquisition time = 429 s) was acquired after administrating first gadolinium injection. Finally, using the slice where the lesion was the largest, dual-echo DCE/DSC gradient-echo was performed (TR = 25 ms, TE_1_ = 1.70 ms, TE_2_ = 8 ms, flip angle = 35°, slice thickness = 3.0 mm, matrix size = 256×216,FOV = 380 mm×380 mm, averages = 1,slices = 1, measurements = 220, acquisition time = 359 s) after second gadolinium injection. 15 minutes elapsed between the first gadolinium injection and second gadolinium injection. For both injections, gadolinium was given through a catheter placed within the antecubital vein at a dose of 0.1 mmol/kg via a power injector at a rate of 3 ml/s, followed by a 10 ml normal saline flush. The total administered dose of gadolinium contrast was 0.2 mmol/kg.

### MR Image Analysis

Image data was saved and transferred to an offline workstation. Custom software written in house with Matlab 7.6 (MathWorks, Natick, Mass, USA) was used for subsequent analysis.

Peak enhancement ratio (PER) and time from contrast agent arrival to peak enhancement (T_max_) are semi-quantitative T_1_W related parameters and they are often used in DCE MRI. Relative regional blood flow and regional flood volume are T_2_*W related parameters (perfusion parameters) and the most relevant parameters obtained in dynamic susceptibility-contrast MRI (DSC MRI). The semi-quantitative T_1_W related parameters, including PER and T_max_ of conventional DCE MRI (c-PER, c-T_max_) and dual-echo MRI (d-PER, d-T_max_), and the T_2_*W related parameters such as rBV and rBFof dual-echo MRI (d-rBV, d-rBF) were calculated[15]–[19]. In the calculating of dual-echo MRI parameters, T_2_* effects were removed in T_1_W related parameters analysis and T_1_ effect would be taken into account in perfusion analysis.

### Statistical Analysis

The parametric variables were compared using one-way ANOVA. Pathology results from tissue sampling were considered the gold standard. The mean and variance were used in this setting. Receiver operating curves (ROC) and the area under the ROC curve (AUROC) were calculated as a descriptive tool to assess the overall discrimination of individual parameters and combined parameters. Sensitivity, specificity and Kappa statistic were used with respect to the diagnostic performance. Analysis was performed with SPSS 19 software (SPSS Inc., Chicago, IL). A *P* value of less than 0.05 was considered to indicate a statistically significant difference.

## Results

### Pathology Results

Of the 38 lesions sampled and analyzed, 20/38 (52.6%) were malignant and 18/38 (47.4%) were benign. Of the 20 malignancies, 14 patients had invasive ductal cancer (moderately or poorly differentiated), 3 patients had invasive lobular carcinoma, 2 patients had ductal carcinoma in situ (DCIS) and one patient had micro papillary carcinoma. Of the 18 benign lesions included 10 fibroadenomas, 4 cyclomastopathy, 3 plasma cell mastitis, and 1 galactoma.

### Comparison of the Semi-quantitative Conventional DCE MRI and Dual-echo MRI Parameters between Breast Cancer and Fibroadenoma

Breast cancers displayed a lower value of T_max_ with conventional DCE MRI (**c-**T_max_) than fibroadenomas (*P* = 0.014). Breast cancers also had lower values of T_max_, rBF and rBV with dual-echo MRI (d-T_max_, d-rBF and d-rBV) than fibroadenomas (*P* = 0.017, *P* = 0.029, *P* = 0.046, respectively). No significant difference regarding peak enhancement ratio (PER) of conventional DCE MRI and dual-echo MRI (*P* = 0.423, 0.252, respectively) was found between breast cancers and fibroadenoma (Table 1).

**Table 1:** Semi-quantitative conventional DCE MRI and Dual-echo MRI parameters of breast lesions.

Parameter	Fibroadenoma (n = 10)	Malignancy (n = 20)	t	p
**c-PER**	1.6(1.12–2.09)	1.44(1.23–1.65)	0.663	0.423
**c-Tmax**	272(234–311) s	219(194–244) s	6.99	0.014*
**d-PER**	0.456(0.303–0.608)	0.612(0.428–0.796)	1.367	0.252
**d-Tmax**	183(173–193) s	168(155–175) s	6.420	0.017*
**d-rBF**	0.015(0.009–0.022)	0.044(0.027–0.062)	5.319	0.029*
**d-rBV**	0.005(0.001–0.008)	0.012(0.007–0.017)	4.306	0.046*

Mean value and 95% confidence interval are given for each parameter.

**Figure 1:**
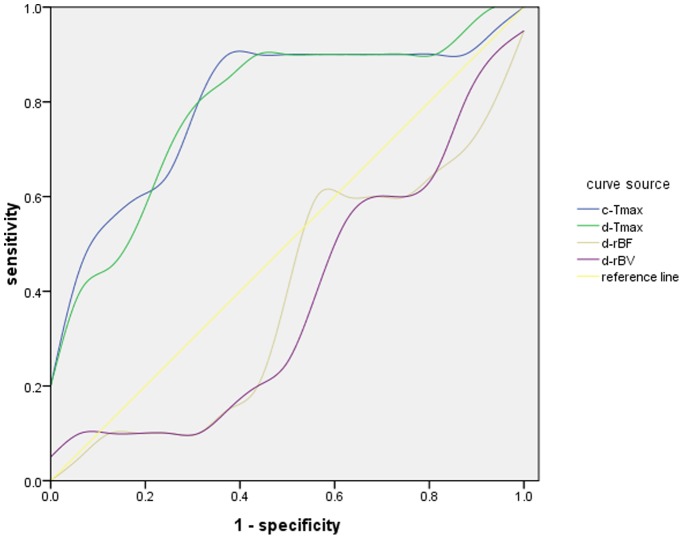
The ROC curves of c-T_max_,d-T_max_,d-rBF and d-rBV.

ROC analysis of both semi-quantitative conventional DCE MRI and dual-echo MRI parameters are given in Table 2, with ROC curves shown in Figure 1. The most relevant factors for discriminating breast cancer from fibroadenoma, based on the areas under the ROC curve (AUROC) were c-T_max_, d-T_max_, d-rBF and d-rBV, (0.800, 0.794, 0.613,0.619, respectively). Using ROC analysis, a c-T_max_<249 s had a sensitivity of 90% and a specificity of 68.8% for predicting malignancy, a d-T_max_<183(s) had a sensitivity of 80% and a specificity of 75% for predicting malignancy, a d-rBF<0.024 had a sensitivity of 50% and a specificity of 80% for predicting malignancy, a d-rBV<0.005 had a sensitivity of 60% and a specificity of 80% for predicting malignancy.

**Table 2:** ROC analysis of the semi-quantitative conventional DCE MRI and dual-echo MRI parameters.

Parameters	AUROC	*p*	Cut-offValue	Sensitivity	Specificity
**c-PER**	0.594 (0.363–0.825)	0.429	1.454	70%	56.3%
**c-Tmax**	0.800 (0.606–0.994)	0.011	249s	90%	68.8%
**d-PER**	0.513 (0.274–0.751)	0.916	0.473	60%	62.5%
**d-Tmax**	0.794 (0.605–0.983)	0.013	183 s	80%	75%
**d-rBF**	0.613 (0.383–0.842)	0.029	0.024	50%	80%
**d-rBV**	0.619 (0.391–0.846)	0.056	0.005	60%	80%

Combining the d-T_max_ of dual-echo MRI with rBV, sensitivity is 90% and specificity becomes 70%. When combining the three factors of T_max_, rBF and rBV, sensitivity remains unchanged at 90% but specificity increases to 80% (Table3).

**Table 3:** Accuracy of combining of T_max_, rBF and rBV of Dual-echo DCE MRI to differentiate breast cancers from fibroadenomas.

Joint Parameters	Sensitivity*	Specificity**	Kappa	*p*
d-T_max_, d-rBF	90% (18/20)	70% (7/10)	0.615	0.001
d-T_max_, d-rBV	85% (17/20)	80% (8/10)	0.634	0.000
d-T_max_, d-rBFand d-rBV	90% (18/20)	80% (8/10)	0.615	0.001

* Data in parenthesis were positive cases tested by imaging vs gold standard.

** Data in parenthesis were negative cases tested by imaging vs gold standard.

## Discussion

Conventional T_1_W DCE MRI typically uses high spatial resolution to estimate T_1_W related semi-quantitative parameters such as c-PER and c-T_max_. Despite high sensitivity, diagnostic specificity remains unsatisfactory [7], [11].The addition of perfusion-related parameters obtained from T_2_*W DSC MRI, such as rBF and rBV[8]–[10], has been shown to increase examination sensitivity and specificity. Improved specificity likely is due in part to an increased number of capillaries and a greater mean capillary diameter in malignant tissues compared to those in benign tissues [7].

It has been shown that simultaneous measurement of T_1_W and T_2_*W parameters can improve the accuracy of both perfusion parameters and permeability kinetics using dynamic contrast enhancement [14]. In particular, T_2_* effects of gadolinium contrast agents, which are more pronounced with increased concentrations, can be measured to reduce underestimation of peak enhancement and overestimation of permeability [14]. A dual-echo gradient echo sequence is a requirement to perform simultaneous T_1_W and T_2_*W measurements using a single contrast dose, and eliminates motion artifact between measurements, since echoes are spaced less than 7 ms apart. Despite the attractiveness of such a technique, dual-echo MRI has not been performed in the breast until now due to various technical challenges[20]–[22].Furthermore, the effects of T_1_-corrected perfusion and T_2_*-corrected T_1_W semi-quantitative parameters have never been evaluated within breast tissue. In fact, there are no comparison studies between normal, benign, or malignant breast with regard to T_2_* effects on routinely-acquired T_1_W dynamic perfusion parameters, and systemic errors can be considerable (particularly within tumors) when this effect is not considered. The dual-echo technique magnetic resonance sequence and analysis software we have implemented leverages multiple recent techniques developed by our group for imaging the breast[23]–[25] to explore the clinical potential of reducing the false positive rate in clinical breast MRI.

The combination of conventional DCE MRI and DSC MRI with two administrations of contrast agent has demonstrated substantially improvement in the diagnostic specificity of breast MRI [7], [11], [12]. Our data show that dual-echo MRI can simultaneously measure the T_1_W and T_2_*W related parameters and improves the accuracy of differentiating breast cancer from fibroadenomas. Dual-echo MRI with a single administration of contrast agent has the advantage of eliminating the expense of a second contrast dose administration, the elimination of a second imaging sequence. Furthermore, the dual-contrast dose technique necessitates a delay between contrast dose administrations to allow washout of residual contrast from the first administration; the dual-echo MRI technique obviates this delay, which typically is 15 minutes. Finally, reduction of total contrast agent administered improves the safety profile of the examination.

This study has several limitations. Only a small number of patients were included, as the intent was an initial investigation into the feasibility and applicability of dual-echo MRI in the human breast. A larger number of patients would permit improved sensitivity, specificity, and accuracy estimations in various subtypes of malignant and benign lesions. Furthermore, our current implementation of dual-echo MRI only allows a single slice to be acquired through a lesion with sufficient temporal and spatial resolution. However, we anticipate that with further iterations in sequence design and improvements in MRI technology a substantially larger imaging volume is achievable.

### Conclusions

In conclusion, we show that dual-echo MRI with a single administration of contrast agent can simultaneously measure both T_1_W and T_2_*W related kinetic and perfusion parameters in the breast. We further show that combining T_1_W DCE MRI measurement of contrast kinetics with T_2_*W DSC MRI perfusion measurements improves the diagnostic performance of breast MRI to differentiate breast cancer from benign fibroadenomas.
